# Long-Term Antimicrobial Performance of Textiles Coated with ZnO and TiO_2_ Nanoparticles in a Tropical Climate

**DOI:** 10.3390/jfb13040233

**Published:** 2022-11-09

**Authors:** Varvara O. Veselova, Vladimir A. Plyuta, Andrei N. Kostrov, Darya N. Vtyurina, Vladimir O. Abramov, Anna V. Abramova, Yury I. Voitov, Darya A. Padiy, Vo Thi Hoai Thu, Le Thi Hue, Dinh Thi Thu Trang, Alexander E. Baranchikov, Inessa A. Khmel, Victor A. Nadtochenko, Vladimir K. Ivanov

**Affiliations:** 1 Kurnakov Institute of General and Inorganic Chemistry, Russian Academy of Sciences, Leninsky Prospekt 31, 119991 Moscow, Russia; 2 N.N. Semenov Federal Research Center for Chemical Physics, Kosygina Street 4, Building 1, 119991 Moscow, Russia; 3 Institute of Molecular Genetics of National Research Centre “Kurchatov Institute”, Akademika Kurchatova Square 2, 123182 Moscow, Russia; 4 Russian-Vietnamese Tropical Research and Technological Center, Nguyễn Văn Huyên Street, Nghĩa Đô Ward, Cầu Giấy District, Hanoi 11307, Vietnam

**Keywords:** climate test, composite materials, metal oxide nanoparticles, ultrasonic cavitation, antimicrobial activity, field testing

## Abstract

This paper reports the results of the large-scale field testing of composite materials with antibacterial properties in a tropical climate. The composite materials, based on a cotton fabric with a coating of metal oxide nanoparticles (TiO_2_ and/or ZnO), were produced using high-power ultrasonic treatment. The antibacterial properties of the materials were studied in laboratory tests on solid and liquid nutrient media using bacteria of different taxonomic groups (*Escherichia coli*, *Chromobacterium violaceum*, *Pseudomonas chlororaphis*). On solid media, the coatings were able to achieve a >50% decrease in the number of bacteria. The field tests were carried out in a tropical climate, at the Climate test station “Hoa Lac” (Hanoi city, Vietnam). The composite materials demonstrated long-term antibacterial activity in the tropical climate: the number of microorganisms remained within the range of 1–3% in comparison with the control sample for the duration of the experiment (3 months). Ten of the microorganisms that most frequently occurred on the surface of the coated textiles were identified. The bacteria were harmless, while the fungi were pathogenic and contributed to fabric deterioration. Tensile strength deterioration was also studied, with the fabrics coated with metal oxides demonstrating a better preservation of their mechanical characteristics over time, (there was a 42% tensile strength decrease for the reference non-coated sample and a 21% decrease for the sample with a ZnO + CTAB coating).

## 1. Introduction

The excessive use of antibiotics causes the emergence of drug-resistant bacterial strains. The search for new effective means of their suppression, to provide long-term antibacterial protection, is a rapidly developing area of research [1]. Metal oxide nanoparticles have been demonstrated to have broad antimicrobial activity against bacteria (both gram-positive and gram-negative), viruses, fungi, and protozoa [2]. Most importantly, their mechanism of action against bacteria differs from the standard mechanisms of antibiotics activity, making it possible to overcome multi-drug resistance [3].

This paper reports on an in-depth study of the antimicrobial performance of composite materials based on cotton fabric with coatings containing ZnO and TiO_2_ nanoparticles, both in the laboratory and in field tests in a tropical climate. These nanoparticles have been thoroughly studied and are known to demonstrate substantial antimicrobial effects [4]. Zinc oxide nanoparticles are an effective antimicrobial agent that is used against different pathogenic microorganisms, such as *Escherichia coli*, *Pseudomonas aeruginosa*, *Staphylococcus aureus*, *Candida albicans*, etc. [5,6,7,8,9]. The antimicrobial activity of ZnO increases for particles of smaller size, larger specific area, and with greater porosity; this is explained by their higher reactivity [5,10,11,12]. The biocidal effect of the TiO_2_ photocatalyst is also well-known from extensive experimental data that demonstrate the feasibility of the photocatalytic disinfection process [13,14]. Composite materials based on titanium dioxide subjected to light excitation effectively destroy both gram-negative and gram-positive bacteria, as well as yeasts [15].

Recent studies have proved that nanoscale metal oxides, including TiO_2_ and ZnO, can also be used as fungicides; for example, these oxides are able to suppress the growth of *Fusarium oxysporum* [16], *Aspergillus niger* [17,18], and *Penicillium expansum* [9].

An important concern for antibacterial coatings is their safety for everyday use [19]. ZnO nanoparticles are environmentally friendly, non-toxic, bio-safe, and biocompatible. The US Food and Drug Administration has listed ZnO, along with four other zinc compounds, as a “generally recognized as safe (GRAS)” substance [20]. Thus, ZnO coatings can be safely used in a humid or unventilated environment, with no health problems. In turn, TiO_2_ is widely used as a component of sunscreens [21,22] and as a food additive [23]. Hence, titanium dioxide can be safely employed as a low-concentration component of a fabric coating [24].

An essential task for the successful biomedical application of titanium and zinc oxides is to create a material with a prolonged and stable antibacterial effect, combined with cytocompatibility and low cytotoxicity. One problem with using antimicrobial inorganic materials is the rapid and uncontrolled release of nanoparticles and ions. The long-term stability of bactericidal materials is crucial for their application [25,26,27]. In order to achieve the long-term and controlled release of antibacterial agents, different ways to immobilize metal oxide nanoparticles in organic or inorganic matrices have been studied extensively in recent years [28].

One possible approach to producing stable fabric coatings is the use of ultrasonic cavitation for the incorporation of oxide nanoparticles in textile materials. This method ensures the uniform distribution of the coating, allows for large-scale production, and provides stability of the coating for at least 20 washing cycles [29,30,31].

While the antibacterial and fungicidal activity of ZnO and TiO_2_ has been studied extensively, along with various proposed methods for oxide-based composite synthesis, there remains a limited amount of data on the performance of the composites in the field. The effect of climate might be extremely significant, and it could severely affect the rate of fabric deterioration [32]. Antibacterial activity depends on temperature [33], humidity [10], and the degree of insolation. A tropical climate is characterized by high temperatures and high humidity. The biological diversity of microorganisms occurring in a tropical climate is significantly greater than that in other climatic zones [34]. Materials intended for use in a tropical climate will be subjected to very harsh environmental conditions, meaning that such materials require special testing.

This paper reports on the whole production cycle of textiles coated with zinc and/or titanium dioxide nanoparticles, and the results of their testing both according to standard laboratory-scale procedures, and in field tests carried out in the tropical climate at the Climate test station “Hoa Lac” (Hanoi city, Vietnam).

## 2. Materials and Methods

### 2.1. Preparation of the Fabric Samples

Two types of aqueous precursor solutions were prepared, with the following compositions: 1) 10 g/L of zinc nitrate hexahydrate (Acros, 98%) and 0.2 g/L of cetyltrimethylammonium bromide (CTAB); 2) 10 g/L of titanyl sulphate (Sigma-Aldrich, St. Louis, MO, USA) and 10 g/L of zinc nitrate hexahydrate (Acros, 98%).

White calico (100% cotton) with a density of 140 g/m^2^ (manufactured by OOO “IvanovoTextile”, Russia) was placed in a precursor solution and treated with ultrasound at 30 ± 2 °C [29,30,35,36]. The frequency of ultrasonic vibrations was 22 kHz, the distance between the ultrasonic emitter and the textile was 20 mm, the volume of the working chamber was 75 L, the total electrical power (4 ultrasonic emitters) was 2.5 kW, the acoustic power was 25–30 W/L, the temperature was kept constant by a heat exchanger. Next, the textiles were removed from the solution, and neutralization was carried out in a desiccator loaded with 3 wt.% solution of NH_3_ (GOST 24147-80). The fabric was washed under running cold distilled water until a neutral pH was achieved. The washed sample was dried at 120 °C for 3 h in a drying cabinet.

Two types of the composite material were produced, based on cotton fabric with a coating of: (1) ZnO nanoparticles with the addition of cetyltrimethylammonium bromide (CTAB); (2) a mixture of ZnO and TiO_2_ nanoparticles. The materials for the field tests were produced using a semi-industrial equipment prototype and a methodology described elsewhere [30].

### 2.2. Preliminary In Vitro Tests

The antibacterial activity of the composite materials was tested using the following model objects: (1) opportunistic bacteria *Escherichia coli* strain BW25113 (the parent strain for the Keio Collection of single-gene knockouts) [37,38]—the strain was kindly provided by Dr. Alexander Mironov (Engelhardt Institute of Molecular Biology, Russian Academy of Science, Moscow, Russia); (2) opportunistic human pathogen *Chromobacterium violaceum* strain CV12472 (a characteristic representative of the soil and water microbiome in tropical and subtropical climatic zones [39])—the strain was kindly provided by Dr. Leonid Chernin (The Hebrew University of Jerusalem, Rehovot, Israel); (3) rhizosphere strain *Pseudomonas chlororaphis* strain 449, which has antagonistic activity against plant-pathogenic fungi and bacteria [40]—the strain was obtained from the rhizosphere of maize (Ukraine) [41].

Two variations of the experimental procedure were used: (a) the growth of bacterial cells on a solid nutrient medium in the presence of textile composites; (b) the growth of bacterial cells in a liquid cultural medium in the presence of textile composites.

Bacteria were grown and maintained on liquid (Miller Luria-Bertani Broth—LB) and solid (LB with 1.5% agar—LA) nutrient-rich media at 30 °C, with stirring (110 rpm), to ensure favorable growth conditions.

#### 2.2.1. Determination of the Antibacterial Effect of Composite Materials on a Solid Nutrient Medium

Bacterial strains were grown in liquid LB medium (5 mL) for 12–16 h at 30 °C, with stirring (110 rpm). Then, 5 mL of 0.5% LA (soft agar) was melted and brought to 45 °C in a water bath, after which 100 µL of culture was added. Petri dishes with 20 mL of 1.5% LA medium were treated with UV for 45 min, then the prepared soft agar containing bacterial culture was added to the surface of 1.5% LA medium. Soft agar was allowed to harden, after which the fabric samples were transferred to the surface of the agar, (two samples of each type of composite material were placed in each Petri dish; the experiment was carried out in two repetitions). The composite materials were cut to be 10 mm × 10 mm (S = 100 mm^2^) in size and pre-treated with UV irradiation for 20 min on each side. The Petri dishes were placed in a thermostat at 30 °C. After 24 h of incubation, fabric samples were moved from the Petri dishes to polypropylene microcentrifuge tubes with 1.5 mL of saline solution (0.85% NaCl) and thoroughly mixed on a vortex. Then, 200 µL of the mixture was poured into a 96-well polystyrene microtiter plate (OAO “Medpolymer”, Saint-Petersburg, Russia—eight wells for each type of fabric coating) and the optical density was measured at λ = 595 nm. The bacterial suspension was also used to make successive dilutions (up to 10^6^ times) in saline solution (100 µL of suspension in 900 µL of saline solution). Then, 10 µL of each dilution was placed on a Petri dish with 20 mL of 1.5% LA. A CFU count was carried out after 24 h of growth at 30 °C.

The antibiotic tetracycline (Tc, 10 mg/mL) was used as a positive control. After 24 h of cultivation at 30 °C, the diameter of the growth inhibition zone of 2 µL Tc applied on a soft agar-containing bacterial culture was 22 ± 1 mm. The diameter of the growth inhibition zone of the control fabric sample (fabric without nanoparticles), after 24 h of cultivation at 30 °C, was 11 ± 1 mm.

#### 2.2.2. Determination of the Antibacterial Effect of Composite Materials in a Liquid Nutrient Medium

Bacterial strains were grown in liquid LB medium (5 mL) for 12–16 h at 30 °C, with stirring (110 rpm). Then, the culture was diluted 100 or 1000 times in fresh LB medium and grown for a further 2 h at 30 °C, with stirring (100 rpm). After 2 h of incubation, 200 µL of the culture was put into a 96-well polystyrene microtiter plate, to enable measurement of the optical density of the culture at λ = 595 nm (the zero point). Then, 5 mL of the culture was put into glass tubes and the composite materials were added. The composite materials were cut to be 10 mm × 10 mm in size and pre-treated with UV for 20 min on each side. Tubes with culture and fabric samples were incubated for another 4 h at 30 °C and then thoroughly mixed on a vortex. After that, 200 µL of the bacterial suspension was poured into a 96-well polystyrene plate and the optical density was measured again. The bacterial suspension was used to make successive dilutions (up to 10^6^ times) in saline solution (100 µL of suspension in 900 µL of saline solution). Then, 10 µL of each dilution was placed on a Petri dish with 20 mL of 1.5% LA. A CFU count was carried out after 24 h of growth at 30 °C.

### 2.3. Field Tests

The fabric composite samples were sterilized at 120 °C for 30 min and then cut into 5 cm × 10 cm pieces. The composite materials were placed on two types of test sites: (a) Concrete site: fabric samples were clamped on a wall with an inclination of 45°, with a minimum distance between the samples of 3 cm, and placed on a solid concrete test site with no shading (for the evaluation of the effect of solar irradiation on composite materials); (b) Mycological site: fabric samples were placed in a cabinet located on a site for microbiological tests in a shaded area. All field experiments were performed at the Climate test station “Hoa Lac” (Hanoi city, Vietnam). Photographs of the two test sites are provided in Figures S1 and S2 (see Supplementary Materials). Weather conditions (temperature T, °C; humidity, %; rainfall, mm; solar irradiation, MJ/m^2^; etc.) at the test sites are listed in Table S1 (see Supplementary Materials).

### 2.4. Isolation of Microorganisms

The fabric composite samples from the test sites were collected after 1, 3, and 4 months of exposure, with microbial strains isolated from the samples. The samples collected were placed in centrifuge tubes with 10 mL 0.85 wt.% NaCl solution and the test tubes were stirred on a shaker for 30 min at a speed of 200 rpm at 30 °C. The resulting solutions were sequentially diluted ten times. Then, 100 µL of the diluted solution was taken, using a pipette, and applied with a spatula to a Petri dish with a nutrient medium on which microorganisms grew for 3 days at 30 °C. The microorganisms were counted in Petri dishes and transferred into separate Petri dishes to isolate pure cultures. The following selective nutrient media were used: (1) Meat Peptone Broth (MPA, for bacteria), g·L^−1^: meat extract 5; peptone 10; NaCl 5; agar 20; pH = 7.0; (2) Hansen (for yeasts), g·L^−1^: glucose 50; KH_2_PO_4_ 3; MgSO_4_·7H_2_O 3; peptone 10; agar 20; pH = 6.0; (3) Gause-1 (for actinomycetes) g·L^−1^: starch 20; MgSO_4_·7H_2_O 3; K_2_HPO_4_ 3; KNO_3_ 1; NaCl 0.5; FeSO_4_·7H_2_O 0.01; agar 20; (4) Potato Dextrose Agar (PDA, for fungi), g·L^−1^: potato extract 200; glucose 20; agar 16.

Hansen, Gause-1, and PDA medium solutions were stirred at 60 °C. Then, chloramphenicol (concentration 0.1 g·L^−1^) was added and the prepared media were poured into Petri dishes.

### 2.5. Identification of Isolated Microorganisms

#### 2.5.1. Identification of Bacteria

DNA isolation, PCR, and electrophoresis were performed according to the G-spin^TM^ Genomic DNA Extraction Kit [for Bacteria] protocol. 16S-rDNA was amplified with primers 27F and 1495R (27F: 5′-GAGAGTTTGATCCTGGCTCAG-3′; 1495R: 5′-CTA CGGCTACCTTGTTACGA-3′ [42]. For the PCR reaction, the following components were used: water 8.5 µL; 27F: 1 µL; 1495R: 1 µL; DreamTaq PCR Master Mix: 12.5 µL; DNA: 2 µL. PCR thermal cycle conditions: initial cycle 94 °C for 5 min and 35 cycles of denaturation at 94 °C for 1 min, annealing 52 °C for 1 min, extension 72 °C for 1.5 min; 72 °C for 5 min. Electrophoresis in agarose gel (1% Redsafe, buffer TBE 1X, 100 V, 40 min) was performed to analyze PCR products.

#### 2.5.2. Identification of Fungi

DNA isolation, PCR, and electrophoresis were performed according to the E.Z.N.A.^®^ Fungal DNA Mini Kit Protocol—Fresh or Frozen—Specimens. PCR primers: ITS1 (5′-TCCGTAGGTGAACCTGCGG-3′) and ITS4 (5′-TCCTCCGCTTATTGATATGC-3′) [43]. PCR components: Water 8.5 µL; ITS1: 1 µL; ITS4: 1 µL; DreamTaq PCR Master Mix: DreamTaq PCR Master Mix: 12.5 µL; DNA: 2 µL. PCR thermal cycle conditions: initial cycle 95 °C for 2 min and 35 cycles of denaturation 95 °C for 30 s, annealing 55 °C for 30 s, extension 72 °C for 60 s; 72 °C for 10 min. Electrophoresis in agarose gel (1.5% Redsafe, buffer TBE 1X, 100 V, 40 min) was performed to analyze PCR products.

### 2.6. Study of the Physical and Mechanical Characteristics of Composite Materials

For the quantitative detection of metal oxides on the textile samples, approximately 1 g of the coated textile was placed in a platinum crucible and ashed at 1000 °C in air.

The phase composition of the mixtures was determined by X-ray phase analysis, using a Bruker D8 Advance diffractometer (Cu_Kα_ radiation, Ni filter, and LYNXEYE detector). The diffraction data were collected in the 2θ angle range from 10 to 70° in 0.02° increments with an accumulation time of 0.2 s/step. Identification of diffraction maxima was carried out using the JCPDS database.

Analysis of elemental composition was performed by energy dispersive X-ray (EDX) spectroscopy using a high-resolution scanning electron microscope (Carl Zeiss NVision 40) equipped with an Oxford Instruments X-Max detector. The analysis was carried out at an accelerating voltage of 20 kV and a working distance of 11 mm. For calibration, the standard metal cobalt reference sample was used.

The surface morphology of the composite materials obtained was studied by scanning electron microscopy (SEM). SEM images were obtained using a Prisma E microscope (Thermo Scientific, Czech Republic) with an accelerating voltage of 3.5 kV. The samples were preliminarily coated with a 10 nm-thick gold layer, using a Q150R ES plus sputter coater (Quorum Technologies, UK).

The contact angle was measured with the help of a Lonroy SDC-350 contact angle measurement system, in ambient conditions; water droplet volume ~5 µL; no tilt.

The tensile strength of the samples was measured according to the TCVN 1754-1986 standard on a device model: Zwick/Roell Z010TH Proline (Germany).

### 2.7. Statistical Analysis

The statistical analysis of the experiments was carried out using IBM SPSS software v. 26 (New York, NY, USA). Significant differences were determined using a one-way analysis of variance (ANOVA), followed by Tukey’s HSD (Honestly Significant Difference) post hoc test. Differences were considered to be significant at *p* ≤ 0.05. All laboratory tests to determine the antibacterial effect of composite materials were performed in two replicates, each with eight technical repetitions. During field testing, all the experiments were carried out in three repetitions.

## 3. Results and Discussion

### 3.1. Production and Characterization of Coated Textiles

Composite materials based on cotton fabric with two types of antibacterial coatings (ZnO + TiO_2_ and ZnO + CTAB) were prepared as described above. Ultrasonic cavitation has previously been proven to be an effective way to immobilize nanoparticles on cotton fabric. The nuances of ultrasonic cavitation technology can be found elsewhere [29,30,31,35].

**Figure 1:**
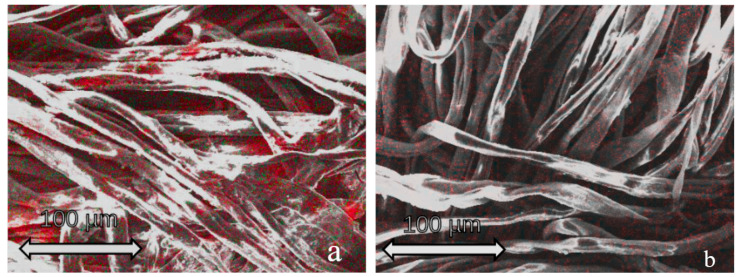
EDX analysis results for samples coated with TiO_2_ + ZnO (**a**) and ZnO + CTAB (**b**). (**a**) Zn content is shown in green, Ti content is shown in red. (**b**) Zn content is shown in red, for better contrast.

The elemental composition of the samples obtained was analyzed using the EDX method (Figure 1). EDX mapping showed a uniform distribution of the coating material on the textile fibers.

Phase composition of the coating was comprehensively analyzed recently [30]. The sediments from the mother liquors after sonication were subjected to XRD analysis. Titanium dioxide was present both as anatase and rutile. Zinc, in both types of coating, was dominantly present as ZnO, although a negligible amount of ZnSO_4_ in the ZnO + TiO_2_ sample was also observed. It seems reasonable to assume that the phase composition of the sediments directly corresponds to the coating composition.

The content of metal oxides in the fabric samples was determined gravimetrically and was found to be 0.9 wt.% for the ZnO + TiO_2_ coating and 0.3 wt.% for the ZnO + CTAB coating. The lower oxide loading in the ZnO + CTAB coating was probably due to the fact that only a limited amount of ZnO can be introduced into the fabric with the ultrasonic cavitation technique. The data obtained agree well with the reported 0.25 wt.% for ZnO and 0.48 wt.% for TiO_2_ for the similarly prepared ZnO + TiO_2_ coating [30]. It can be concluded, with some confidence, that in the current work the same ZnO/TiO_2_ ratio in the ZnO + TiO_2_ sample was obtained.

**Figure 2:**
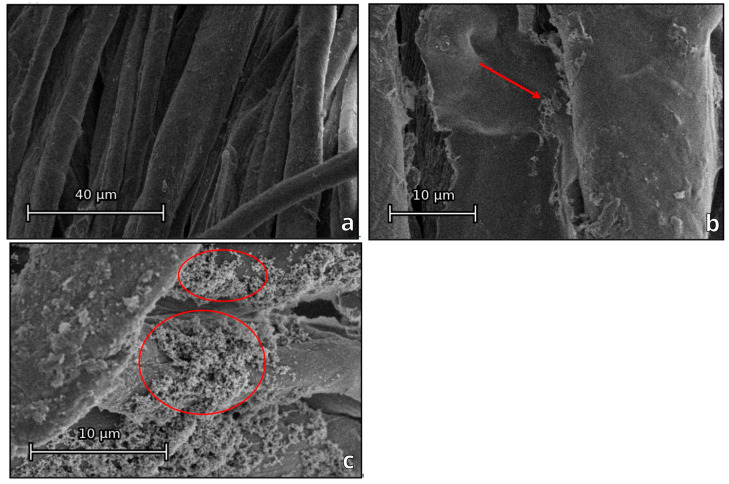
SEM images of the original cotton fabric (**a**), fabric coated with the composition ZnO + CTAB (**b**) and ZnO + TiO_2_ nanoparticles (**c**).

The nanostructure and surface properties of the material strongly affect the reactivity of the coating, e.g., nano-TiO_2_ generally produces many photo-generated reactive species responsible for either direct or indirect inactivation of microorganisms [44]. The importance of nanotexturization of a surface, as well as its morphology in terms of antibacterial properties, has previously been shown for a number of materials [45,46]. The surface morphology of the samples obtained was studied using SEM (Figure 2). In the case of the ZnO + CTAB coating, a significant amount of surfactant remained on the cotton fibers, resulting in a relatively smooth coating (Figure 2b, remains of the surfactant on the fiber are shown with an arrow). The deposition of titanium dioxide nanoparticles on the textile resulted in the formation of a nanotextured surface (Figure 2c, areas with nanotexture are given in red circle).

**Figure 3:**
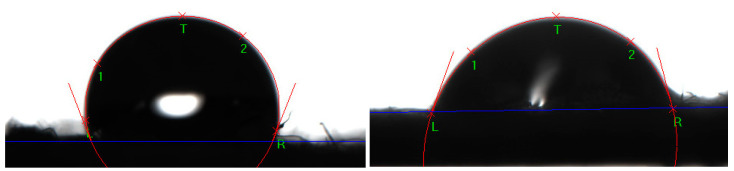
Contact angle measurement for the fabric with a ZnO + TiO_2_ coating (**left**) and a ZnO + CTAB coating (**right**).

Such a texture is known to contribute to bacteria elimination through the mechanical destruction of membranes [47]. The nanotexture also imparts a certain degree of hydrophobicity (Figure 3): the water contact angle for the fabric with ZnO + TiO_2_ was measured to be 112°. A hydrophobic surface has less bacterial adhesion [48], thus reducing the number of bacterial species to be eliminated through chemical interaction. In case of the ZnO+CTAB coating the contact angle was 73°.

### 3.2. Evaluation of Antibacterial Activity in Laboratory Tests

Standard laboratory tests were carried out to assess the antibacterial efficiency of composite materials with ZnO + CTAB and ZnO + TiO_2_ coatings. The results of in vitro tests of the composites against bacteria of different taxonomic groups are presented in Table 1.

On the solid nutrient medium, both coatings exhibited antibacterial activity against tested bacterial strains (*C. violaceum* CV12472, *E. coli* BW25113, and *P. chlororaphis* 449). In the case of the *C. violaceum* CV12472 and *E. coli* BW25113 strains, the effect was less pronounced for the fabric coated with the ZnO + TiO_2_ composition than it was for the fabric coated with the ZnO + CTAB composition. In the case of the *P. chlororaphis* 449 strain, both coatings exhibited almost the same effectiveness in suppressing bacterial growth. CTAB has previously been shown to be an effective antibacterial fabric coating by itself [49]. The antibacterial action of quaternary ammonium compounds (QACs) is attributed to the positive charge, which forms an electrostatic bond with negatively charged sites on microbial membranes [50]. Those electrostatic bonds create stress in the wall, leading to the lysis and death of microorganisms. QACs also cause the death of bacteria by protein denaturation, the disruption of membrane permeability, and the reduction of the normal uptake of nutrients [51,52]. Cetyltrimethyl ammonium bromide (CTAB) is a QAC that is considered to rupture the membrane. Its site of action has been suggested to be the lipid components of the membrane; their damage causes cell lysis [53,54]. These compounds demonstrate both antimicrobial, and antifungal, activity [55]. Hence, the ZnO + CTAB composition showed a high level of efficiency.

No effect of the composites on the growth of bacteria was observed in a liquid medium. Overnight incubation of the fabric samples with the 1000x diluted culture of *C. violaceum* CV12472 resulted in a slight increase in CFU count. It is known that certain concentrations of zinc ions, as well as some other metals, are necessary to maintain cellular metabolism; zinc participates in many cellular processes [56,57]. The observed effect can be explained by the fact that the fabric samples might release zinc ions into the nutrient medium in a concentration that is favorable for bacterial growth. It should also be noted that bacterial cultivation was carried out on nutrient-rich media. The effects of the studied composite materials on the viability of tested bacteria might have been more evident on nutrient-poor media, and this requires further research.

The data obtained demonstrate that the effect of metal oxide coatings depends on cultivation conditions, which should be taken into consideration in practice. The fabrics cannot be used in a liquid nutrient-rich medium, but they effectively suppress the growth of microorganisms in air, and thus might be used outdoors.

### 3.3. Field Tests

#### 3.3.1. The Number of Microorganisms on the Surface of Textiles

Field tests were carried out at the Climate test station “Hoa Lac” to evaluate the outdoor antibacterial activity of composite materials. The results of the field tests (on the open concrete and the mycological sites) are summarized in Table 2.

The number of colonies per sample in the control sample (original cotton fabric without a coating) rapidly increased during exposure to the tropical environment. In contrast, the number of colonies in the coated samples increased slowly over time, proving the inhibitory ability of the composite materials. In general, composite materials had 35–50 times less CFU per sample than the control (see Table 2). The number of different types of microorganisms on the samples after field testing is listed in Table 3.

Composite material samples had fewer microbial species on their surface after field testing than the control samples. The textile with the ZnO + CTAB coating demonstrated very different results in the two test sites: in the mycological site, this coating was the most effective, but on the samples placed on the open concrete site, the number of microorganisms increased over time (Table 2). The surfactant CTAB probably decomposed under direct sunlight, over time, in the open concrete site [58]. QACs have also been shown to undergo biodegradation when treated with mould fungi [59] and, given the prevalence of fungi species at the open concrete site (Table 3) that might be another reason for the loss of coating efficacy.

#### 3.3.2. Types of Microorganisms on the Surface of Textiles

As can be seen from Table 3, the ratio of the microorganism types on the fabric did not change significantly over time. Yeasts were the least common organisms, both on the reference sample and on the coated textiles. The data obtained show that textiles with ZnO + CTAB and ZnO + TiO_2_ coatings were more effective against contamination by bacteria than against fungi, compared with the reference sample in the open concrete site test. Conversely, composite material samples were more effective against contamination by fungi than against bacteria, compared with the reference sample at the mycological test sites. The difference can not be explained by different starting conditions: reference samples at both test sites were found to have very similar proportions and amounts of various microorganisms. As discussed previously, the photocatalytic activity of the coatings plays a major role in their antibacterial properties. Hence, it is logical that antibacterial effects were more prominent at the test site with better insolation. Researchers also, however, attribute the antifungal properties of metal oxide nanoparticles to the formation of reactive oxygen species (ROS) under irradiation [10,60,61]. It has also been demonstrated that the mechanism of nanoparticle action might be different for different types of fungi [9]. Perhaps on the mycological test site, non-ROS-related mechanisms of fungicidal action became prevalent. Further research is required to determine the reasons for the observed changes in microorganism composition.

After 4 months of exposure in a tropical environment, the textile samples were collected and processed, to isolate the strains of microorganisms. In total, 37 strains were isolated: 25 strains of mycelium, four strains of yeasts, and eight strains of bacteria. These data demonstrate that mycelium species were more abundant and can significantly contribute to material deterioration in tropical conditions. Thus, solutions for protecting materials in a tropical climate should focus not only on antibacterial properties, but also on fungicidal activity.

#### 3.3.3. Identification of the Most Commonly Found Microorganisms

Of the 37 isolated strains, the ten most commonly found were identified using the PCR technique. The microorganisms identified are listed in Table 4.

Among the microorganisms identified, there are four species of bacteria, five species of fungi, and one yeast. Gram-positive bacteria (*E. indicum*, *B. paraconglomeratum*, and *B. amyloliquefaciens*) prevailed on the surface of textile materials with metal oxide coatings. Of the four identified species, three have antibiotic, antifungal, or other activity: *E. indicum* has anti-quorum sensing activity [62,63]; *B. paraconglomeratum* can produce biosurfactant with broad antibiotic activity [64]; *S. rhizophila* is a gram-negative bacterium that has been shown to produce volatile antifungal compounds [65]. The fourth, *B. amyloliquefaciens*, is considered to be a plant growth-promoting rhizobacterium (PGPR) and is used to fight root pathogens and improve root tolerance to salt stress [66,67]. The bacterial species identified cannot be considered to be harmful.

The fungi species, however, are more concerning. *C. tenuissimum* is an endophytic fungus, that has been reported to cause plant infections in tropical regions (e.g., leaf spot on carnations, alfalfa, castor, etc., skin sooty and decay disease in pears, dry rot in tomatoes, leaf spot on bananas and leaf blight in watermelons and cucumbers; the fungus is also a hyperparasite of several rust fungi) [68,69]. *S. implicatum* are phytopathogens, and others are considered to be humans’ and animals’ opportunistic pathogens [70]. Strains of *F. solani* filamentous fungus (including *F. solani-melongenae*) are ubiquitous in soil and play a role in decaying plant material, acting as decomposers. They might contribute to the decay of cotton fabric. They are also host-specific phytopathogens and cause plant diseases in some agriculturally important crops (e.g., peas, cucurbits, sweet potatoes, etc.). Moreover, they are increasingly associated with opportunistic infections in humans and animals [71]. *A. sydowii* is a saprophytic fungus distributed worldwide, which occurs in different environments. Besides its terrestrial appearance in soil, it can grow in the sea, due to its salt tolerance. In addition, *A. sydowii* can contaminate food and it occasionally causes human infections; it causes epizootic infections of sea fan corals in marine ecosystems [72]. The fungus *A. wentii* is a widely distributed common soil fungus with a cosmopolitan distribution; it is primarily found in subtropical regions. It has been shown that *A. wentii* infests crops (e.g., corn, cereals, peanuts, etc.) and can contaminate foods, adversely impacting animal and human health [73,74].

The yeast *H. sinensis* (*Bullera sinensis*) has been described on the basis of a strain obtained from a wheat leaf (*Triticum* sp.) in China; it has later been found to occur widely in the tissue and leaf surface of various plants (e.g., rice, corn, sugarcane, elephant grass, and cactus). *H. sinensis* has also shown the ability to produce a large amount of phytohormone indole-3-acetic acid [75].

Thus, the data obtained provide an insight into the species and genus composition of microorganisms identified as frequently occurring on the composite materials tested. Among the species frequently found on the surface of the textile coated with ZnO + TiO_2_ and ZnO + CTAB compositions, it is the fungi species that raise more concern than the bacteria.

### 3.4. Tensile Strength Evaluation

**Figure 4:**
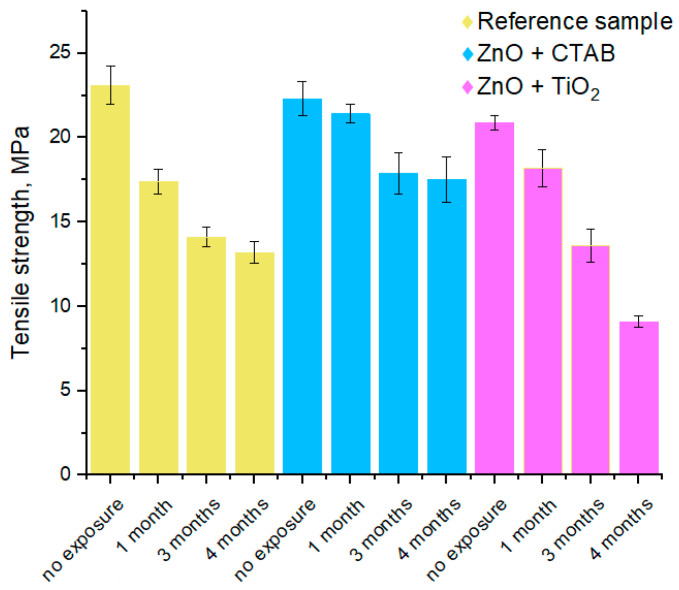
Tensile strength of the fabric samples, after different durations of exposure, on the open concrete site. The standard deviation is the average from three separate experiments.

To provide a rough estimate of the degree of deterioration, the tensile strength of the textile samples was evaluated after different durations of exposure to a tropical environment, following the TCVN 1754-1986 standard protocol. Samples from the open concrete site were chosen for the test, because, according to the data in Table 2 and Table 3 (CFU count and variety of microorganism types), these conditions were the most severe. The results of the measurements are summarized in Table 5 and presented graphically in Figure 4.

The application of ZnO nanoparticles has previously been reported to affect the mechanical properties of a textile (batik) by increasing its tensile strength and decreasing its elongation at break [76]. In the current work, this effect was not observed.

After 4 months of exposure on an open concrete site, the decrease in tensile strength of the reference sample reached 43%. The authors consider this change to be partly caused by the activity of microorganisms acting on the samples and partly caused by weather conditions; (more detailed information about weather conditions is presented in Table S1, see Supplementary Materials). The ZnO + TiO_2_ coated fabric showed a 57% decrease in tensile strength, which was the largest decrease among the fabrics tested. The photo-generated reactive species produced by nano-TiO_2_, while suppressing bacterial growth, are also likely to have oxidized the cotton fibers of the fabric. This effect can be even more significant than that of the activity of microorganisms. The textile with the ZnO + CTAB coating demonstrated the best preservation of mechanical properties, with a decrease of only ~22%. This deterioration can be explained by environmental factors that directly affected the sample (solar radiation, rain, wind, etc.), which led to the destruction (aging) of the fabric structure, changing the properties and durability of the fabric.

Thus, the data obtained on antimicrobial activity, the microbial species found on the samples, and the mechanical characteristics of fabrics coated with metal oxide nanoparticles in a tropical climate, after field testing on open concrete and mycological sites, clearly demonstrate the potential for use of the textile composite materials in a tropical environment. Particular attention must be paid to the antifungal properties of materials intended for use in tropical conditions.

## 4. Conclusions

Textiles with ZnO + CTAB and ZnO + TiO_2_ coatings were produced using an ultrasonic cavitation technique. The surface morphology of the coatings was studied and the ZnO + TiO_2_ composition was shown to exhibit a nanotexture and hydrophobicity. The study of the antibacterial properties of these composite materials, using standard laboratory tests, showed they were not effective in a liquid medium, but that they suppressed more than 50% of bacterial species in the solid nutrient medium than the reference sample. Field tests of the coated fabrics, carried out at the “Hoa Lac” test site (Hanoi city, Vietnam), showed that the coated fabrics reduced the number of microorganisms to 1–3% compared with the reference, both under direct sunlight and at the shaded test site. The textile with a ZnO + CTAB coating lost its efficacy under direct sunlight over time, probably due to the UV-induced decomposition of CTAB. The textile with a ZnO + TiO_2_ coating retained great efficiency at both of the test sites, but a large decrease in tensile strength was observed for this coating under direct sunlight, over time (57%). Future work will focus on testing coated textiles under different climatic conditions (e.g., on the coast, in higher humidity). The coating compositions might be applied to other commonly used fabrics, such as viscose.

The types of microorganisms found on the textile samples were identified, with fungi being the most prevalent species. Bacteria and yeast were either harmless or possessed antibiotic or antifungal properties. Meanwhile, fungal species detected were quite pathogenic. Future research must focus on the development of coatings with pronounced antifungal activity. Overall, the coating compositions tested were effective in the suppression of microorganisms in shaded areas.

## Figures and Tables

**Table 1:** Antibacterial activity of the textiles coated with ZnO + CTAB or ZnO + TiO_2_ compositions. The number of viable microorganisms (CFU) in the reference sample is taken as 100%. In the Table, the numbers represent the decrease in the number of CFU compared with the reference sample; «+» slight stimulation of bacterial growth was observed (no more than 10% of the number of CFU in the reference sample). The different uppercase letters (^a^, ^b^, or ^c^.) above the numbers indicate significant differences (*p* ≤ 0.05; Tukey’s HSD test).

Bacteria Sample	*C. violaceum* CV12472			*E. coli* BW25113		*P. chlororaphis* 449 *
	Solid Medium	Liquid Medium		Solid Medium	Liquid Medium	Solid Medium
		Dilution of Bacterial Overnight Culture (Times)			Dilution of Bacterial Overnight Culture (Times)	
		×100	×1000		×1000	
Reference sample **	<10% ^a^	<10% ^a^	<10% ^a^	<10% ^a^	<10% ^a^	<10% ^a^
ZnO + CTAB	>50% ^b^	<10% ^a^	+ ^a^	>50% ^b^	<10% ^a^	30–45% ^b^
ZnO + TiO_2_	10–20% ^c^	<10% ^a^	+ ^a^	20–50% ^c^	<10% ^a^	45–50% ^c^

* For the strain *P. chlororaphis* 449, the number represents the decrease in biomass concentration (optical density of culture) (as a percentage) that was determined spectrophotometrically by measuring light absorbency at 595 nm. **—original cotton fabric without a coating.

**Table 2:** Results of the field tests of the composite textile materials with metal oxide coatings at the Climate test station “Hoa Lac” (Hanoi city, Vietnam). The average value of CFU from three repetitions is given.

		Duration of Exposure to a Tropical Environment, Months					
Type of Test Site	Type of Coating	1		3		4	
		Strains/Sample	Colonies/Sample (CFU)	Strains/Sample	Colonies/Sample (CFU)	Strains/Sample	Colonies/Sample (CFU)
Concrete open test site	Reference sample *	15	9,600,000	15	11,130,000	15	12,200,000
	ZnO + CTAB	8	95,000 (1% **)	9	305,000 (2.7% **)	9	650,000 (5.3% **)
	ZnO + TiO_2_	10	210,000 (2.2% **)	9	246,000 (2.2% **)	8	225,000 (1.8% **)
Mycological shelf test site	Reference sample *	14	5,950,000	14	8,140,000	14	11,650,000
	ZnO + CTAB	6	167,000 (2.8% **)	6	135,000 (1.7% **)	7	91,000 (0.8% **)
	ZnO + TiO_2_	7	154,000 (2.6% **)	7	153,000 (1.9% **)	8	177,500 (1.5% **)

* original cotton fabric without a coating; ** in comparison with control.

**Table 3:** Number of different types of microorganisms on the composite textile materials with metal oxide coatings after different periods of exposure at the Climate test station “Hoa Lac”(Hanoi city, Vietnam). The average value from three repetitions is given.

Time of Exposure		Number of Species After								
		1 Month			3 Months			4 Months		
Type of Microorganism		Bacteria	Yeast	Fungi	Bacteria	Yeast	Fungi	Bacteria	Yeast	Fungi
Concrete open test site	Reference sample	5	3	7	5	3	7	5	3	7
	ZnO+ CTAB	2	2	4	2	2	5	2	2	5
	ZnO + TiO_2_	2	3	6	2	3	5	2	3	5
Mycological shelf test site	Reference sample	4	3	7	4	3	7	4	3	7
	ZnO + CTAB	2	1	3	2	1	3	3	1	3
	ZnO + TiO_2_	3	2	3	3	3	3	3	3	3

**Table 4:** List of microorganisms that most frequently appeared on the composite textile materials with metal oxide coatings after 4 months of exposure at the Climate test station “Hoa Lac” (Hanoi city, Vietnam).

Species	Strain	Agreement with Genbank Database
Bacteria	*Stenotrophomonas rhizophila*	100%
	*Exiguobacterium indicum*	99.86%
	*Brachybacterium paraconglomeratum*	99.93%
	*Bacillus amyloliquefaciens*	99.72%
Yeast	*Hannaella sinensis*	97.02%
Fungi	*Cladosporium tenuissimum*	100%
	*Sarocladium implicatum*	100%
	*Aspergillus sydowii*	100%
	*Aspergillus wentii*	100%
	*Fusarium solani-melongenae*	100%

**Table 5:** Tensile strength of the composite textile materials with metal oxide coatings after different periods of exposure at the Climate test station “Hoa Lac” (Hanoi city, Vietnam). The average value from three repetitions is given.

Type of Exposure	Type of Coating	Duration of Exposure to the Tropical Environment			
		0 (Before the Field Test)	1 Month	3 Months	4 Months
		Tensile Strength, MPa			
Concrete open test site	Reference sample, original cotton fabric without a coating	23.1	17.4	14.1	13.2
	Fabric coated with ZnO nanoparticles + CTAB	22.3	21.4	17.9	17.5
	Fabric coated with ZnO + TiO_2_ nanoparticles	20.9	18.2	13.6	9.1

## Data Availability

Data is contained within the article or Supplementary Materials.

## Supplementary Materials

The following supporting information can be downloaded at: https://www.mdpi.com/article/10.3390/jfb13040233/s1, Table S1. Weather conditions at “Hoa Lac” (Hanoi, Vietnam); Figure S1. Concrete test site; Figure S2. Mycological test site.
