# Supplementary material for: Long-Term Antimicrobial Performance of Textiles Coated with ZnO and TiO2 Nanoparticles in a Tropical Climate
Source: J Funct Biomater. 2022 Nov 9;13(4):233. doi: 10.3390/jfb13040233 (PMC9680289; doi:10.3390/jfb13040233)
Supplement: Supplementary file 1 [file jfb-13-00233-s001.zip › jfb-2002354-supplementary.pdf]

Table S1. Weather conditions on Hoa Lac (Hanoi, Vietnam).

Month	T, °C					Humidity, %					Rainfall, mm	Total solar irradiation, MJ/m^2	UV (A/B) Average per hour 6^{00} - 18^{00} MJ/m^2
	Monthly average	Monthly average min	Monthly average max	Abs. min	Abs. max	Monthly average	Monthly average min	Monthly average max	Abs. min	Abs. max			
January	20	16	25	14	31	84	72	93	51	95	53,3	190	8,5 (7,5; 0,4)
February	19	14	24	13	28	81	47	90	31	95	26,4	265	14,1 (13,0; 0,4)
March	23	21	26	15	30	86	76	91	47	95	82,8	234	10,8 (9,8; 0,4)
April	22	20	25	14	31	82	70	91	42	94	144,8	352	17,8 (16,5;0,5)
May	28	23	32	19	38	85	70	96	50	100	179,3	410	26,1 (24,8;0,5)
June	30	26	33	23	39	79	60	94	40	100	315,6	621	39,8 (38,3;0,6)
July	30	27	33	22	38	81	69	91	48	100	162,5	536	25,9 (25,5;0,6)
August	29	25	32	23	38	90	80	98	52	100	1020,8	410	29,9 (28,5;0,6)
September	29	24	31	23	38	89	77	99	51	100	591,9	382	26,6 (25,3;0,5)
October	24	21	29	16	34	88	71	98	40	100	491,1	258	19,5 (18,3;0,4)
November	23	20	26	15	31	87	66	99	38	100	112,3	268	18,2 (17,1;0,4)
December	23	20	26	15	31	85	67	98	41	100	12,8	211	13,9 (12,8; 0,4)
Average	**25**					**85**							
Total											**3193,6**	**4137**	**251,1**

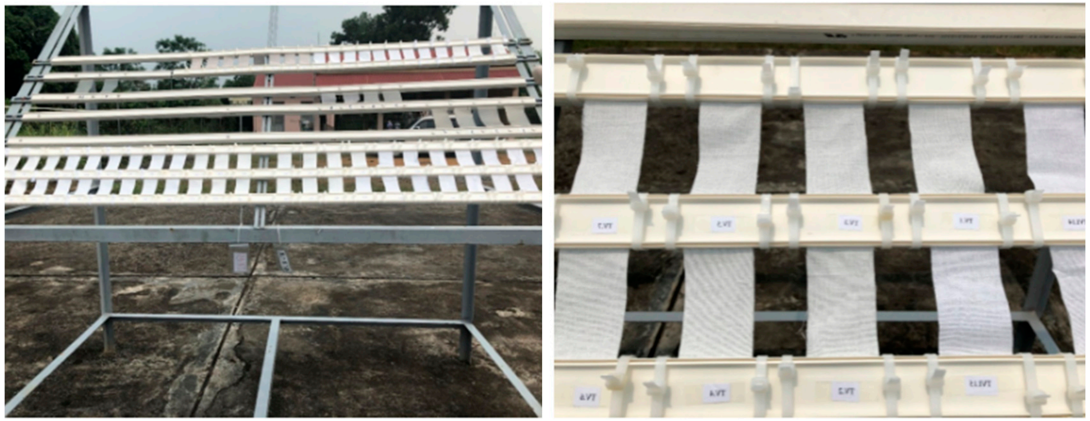

Figure S1. Concrete test site.

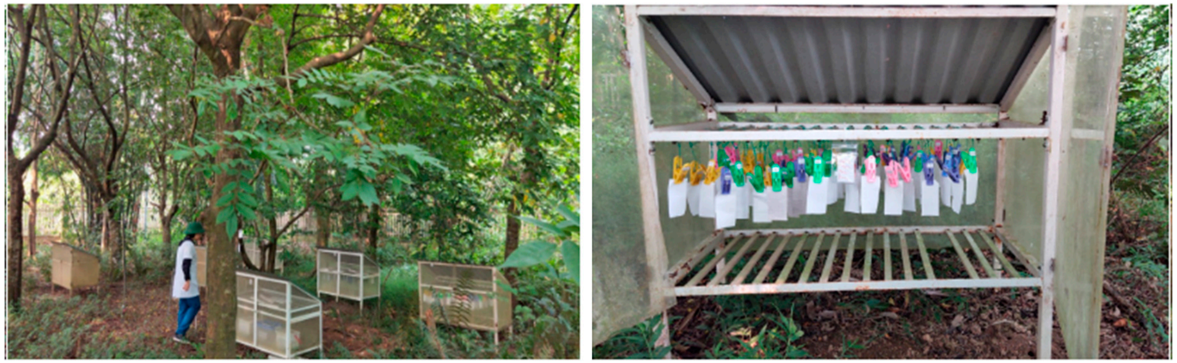

Figure S2. Mycological test site.
